# Supplementary material for: Mental health in medical and biomedical doctoral students during the 2020 COVID-19 pandemic and racial protests
Source: eLife. 2022 Sep 6;11:e69960. doi: 10.7554/eLife.69960 (PMC9448322; doi:10.7554/eLife.69960)
Supplement: Source data 1. [file elife-69960-data1.pdf]

```
/* PHQ */
/* MODEL 1 (FULL MODEL) */;
proc logistic data=_model_;
  model phq_dum=md_phd year_dum gender urm_nom md_phd*year_dum
               md_phd*gender md_phd*urm_nom year_dum*gender
               year_dum*urm_nom gender*urm_nom
               md_phd*year_dum*gender md_phd*year_dum*urm_nom
               year_dum*gender*urm_nom md_phd*year_dum*gender*urm_nom;
  contrast "all interactions" md_phd*year_dum 1, md_phd*gender 1,
                              md_phd*urm_nom 1, year_dum*gender 1,
                              year_dum*urm_nom 1, gender*urm_nom 1,
                              md_phd*year_dum*gender 1,
                              md_phd*year_dum*urm_nom 1,
                              year_dum*gender*urm_nom 1,
                              md_phd*year_dum*gender*urm_nom 1;
  contrast "4-way" md_phd*year_dum*gender*urm_nom 1;
  contrast "all 3-way" md_phd*year_dum*gender 1,
                       md_phd*year_dum*urm_nom 1,
                       year_dum*gender*urm_nom 1;
  contrast "all 2-way" md_phd*year_dum 1, md_phd*gender 1,
                       md_phd*urm_nom 1,year_dum*gender 1,
                       year_dum*urm_nom 1, gender*urm_nom 1;
  contrast "marginal effects" md_phd 1, year_dum 1, gender 1, urm_nom 1;
run;
/* MODEL 2 */
proc logistic data=_model_;
  model phq_dum=md_phd year_dum gender urm_nom
                md_phd*year_dum md_phd*gender;
  contrast "Marginal Effects" md_phd 1, year_dum 1, gender 1, urm_nom 1;
  contrast "Two interactions" md_phd*year_dum 1, md_phd*gender 1;
run;
/* MODEL 3 */
proc logistic data=_model_;
  model phq_dum=md_phd year_dum gender urm_nom
               md_phd*year_dum md_phd*gender
               gender*urm_nom;
  contrast "Marginal Effects" md_phd 1, year_dum 1, gender 1, urm_nom 1;
  contrast "Two interactions" md_phd*year_dum 1, md_phd*gender 1;
  contrast "Gender*URM" gender*urm_nom 1;
run;
/* MODEL 4 */
proc logistic data=_model_;
  model phq_dum=md_phd year_dum gender urm_nom
               md_phd*year_dum md_phd*gender
               year_dum*gender year_dum*gender*md_phd;
  contrast "Marginal Effects" md_phd 1, year_dum 1, gender 1, urm_nom 1;
  contrast "Two interactions" md_phd*year_dum 1, md_phd*gender 1;
  contrast "Additional Effects" year_dum*gender 1,
                                year_dum*gender*md_phd 1;
run;
```
